# Examining the non-linear relationship between sugar consumption and anxiety symptoms in UK biobank data

**DOI:** 10.1186/s12937-025-01277-4

**Published:** 2026-01-26

**Authors:** Xue Yang, Agassi Chun Wai Wong, Qian Li, Hannah Xiaoyan Hui, Liping Zhang, Samuel Yeung-shan Wong

**Affiliations:** 1 Jockey Club School of Public Health and Primary Care, Faculty of Medicine, The Chinese University of Hong Kong, Hong Kong, China; 2 School of Biomedical Sciences, Faculty of Medicine, The Chinese University of Hong Kong, Hong Kong, China; 3 Massachusetts General Hospital, Harvard Medical School, Harvard University, Boston, USA

**Keywords:** Emotional health problems, Total sugar, Glucose, Fructose, Maltose, Sucrose

## Abstract

**Purpose:**

Generalized anxiety disorder (GAD) is a common psychiatric condition. The role of sugar in emotional health is becoming more apparent. This cross-sectional study investigated the potential non-linear associations of sugar on GAD and identified thresholds that would be associated with GAD if these non-linear associations were significant, using the UK Biobank.

**Method:**

A sample of 84,087 subjects was included. Total energy and sugar consumption were calculated using Oxford WebQ. Total sugar, glucose, fructose, maltose, and sucrose as dietary exposure. The Generalized Anxiety Disorder-7 (GAD-7) questionnaire was used to measure anxiety symptoms. The non-linear relationship between sugar and GAD scores was examined using generalized additive models (GAMs).

**Results:**

Significant non-linear relationships were found between sugar consumption and GAD score, and were modified by gender and age. Total sugar and sucrose consumption demonstrated non-linear associations with GAD scores among those aged 45 or younger. In those aged 46 to 64 years, non-linear associations of GAD score were found in total sugar consumption in both genders. Additionally, in females, non-linear associations were also observed across all the sugar types; compared to males, only sucrose consumption showed a significant association. Specifically, the association between sucrose consumption and GAD score followed a J-shaped pattern in both genders. Only sucrose consumption demonstrated a non-linear association with GAD scores in females aged 65 or above.

**Conclusion:**

This study identified non-linear and dose-dependent associations between various types of sugar on anxiety in different gender and age groups, which may have implications for lifestyle psychiatry.

**Supplementary Information:**

The online version contains supplementary material available at 10.1186/s12937-025-01277-4.

## Introduction

Anxiety disorders, characterized by feelings of anxiety and fear [1], are some of the most common psychiatric conditions affecting approximately 3.8% of the global population (GBD 2019 [2]). Anxiety disorders are associated with worsened medical conditions (e.g., cardiovascular diseases and gastrointestinal diseases), increased healthcare utilization, and significant impairments in social, educational, and occupational functioning [3]. A systematic review revealed that anxiety disorders significantly increase healthcare costs at the individual level [4]. Generalized anxiety disorder (GAD) is the most prevalent anxiety disorder in the primary care setting with a high prevalence of 8% [5]. The self-reported prevalence ranges from 17.9% to 28.6% globally [6].

The World Health Organization (WHO) recommends limiting the consumption of free sugar to 10% of total energy intake and a further reduction to 5% is desired [7]. However, the recommendation was based on a dental health and free sugar cohort without differentiating the types of sugars. Little is known about the recommended amount of dietary sugar consumed for mental/emotional health. A recent systematic review [8] only identified only 10 cross-sectional studies and inconsistent findings regarding the associations of various types of sugars on anxiety were reported. For example, four studies suggested positive correlations between added sugar consumption and anxiety [9–12]. Four studies on the associations between the consumption of sugar-sweetened beverages and anxiety showed contradictory findings [13–16]. Three studies reported nonsignificant correlations between sweet consumption and anxiety [13, 16, 17].

Lifestyle psychiatry has suggested that diet and nutrition are modifiable lifestyles and emerging factors in emotional health [18]. For example, some intervention studies reported small beneficial effects of glucose ingestion on mood [19]. Some studies have suggested that sugar and sweets help the brain to produce surges of dopamine, which are widely consumed to reduce anxiety and negative mood [20, 21]. The consumption of high-sugar foods likely attenuates the psychological (anxiety, depressed mood) effects of stress via actions in the periphery (i.e., glucocorticoid receptor signalling in adipose tissue) and in the brain (i.e., plasticity in brain reward regions) [22]. Furthermore, some complex carbohydrates are fermented by gut microbes, producing short-chain fatty acids that appear to have anti-inflammatory and protective effects [23].

On the other hand, while dietary sugar is an essential macronutrient for brain function [24] it can also exist in various forms and has been recognized as a contributor to chronic inflammation [25]. Animal studies have suggested that high sucrose and fructose contents could alter emotional processing and modify behaviours [20, 26, 27], but there is little evidence on other types, such as glucose and maltose. These results may suggest that different types of sugars may play different roles in anxiety. In addition, previous studies on other dietary lifestyles (e.g., alcohol consumption) reported consistent U-shaped relationships with mental/emotional health problems, including anxiety [28, 29]. Both dietary sugar and alcohol are similar in that they are both sources of energy and addictive; evidence has shown that alcohol can activate the brain reward system, but excessive use increases dependence, stress, and physical harm [30, 31]. A similar process could be expected for sugar consumption. Thus, a moderate amount of sugar intake may improve emotions and moods, while further intake may worsen anxiety. No study has tested such nonlinear relationships or identified the threshold.

### The present study

The current study aimed to investigate the potential nonlinear associations of different types of sugars with GAD and identify thresholds of different types of sugars that were linked to GAD if the nonlinear associations were significant. It is hypothesized that 1) the nonlinear associations of different types of sugar consumption on GAD would be statistically significant and that 2) various types of sugar would have different associations with GAD. Since emerging evidence has suggested that added sugars may increase anxiety [8] and other health problems [32], it is essential to investigate the relationship between added sugars and anxiety symptoms, while also considering the various types of sugars involved. Moreover, gender and age are associated with sugar consumption [33], moderate dietary behaviour, and mental health status [34, 35] due to variations in digestion and absorption ability, inflammation, dietary coping, and emotional response. Thus, it would be appropriate to investigate whether sex and age moderate these associations. Investigating the relationship between sugar consumption and anxiety is important because anxiety is highly comorbid with other mental disorders and chronic diseases, and understanding this relationship may help to mitigate anxiety through dietary modifications and further reduce health burdens.

## Methods

### Participants and data collection

The UK Biobank is a prospective cohort study of more than 500,000 participants aged between 40-69 years who were recruited across the UK between 2006 and 2010. A detailed protocol is available online (UK [36]). This study utilizes a cross-sectional design with 24-hour dietary records collected between 2009 and 2012, and the 7-item Generalized Anxiety Disorder data were collected in 2017. For the current study, participants who completed two or more web-based dietary questionnaires for the assessment of previous 24-hour dietary records (Oxford WebQ) between 2009 and 2012 [37] were selected. The exclusion criteria were as follows: 1) missing or incomplete 7-item Generalized Anxiety Disorder data from 2017, and 2) extreme values for total energy intake (e.g., outside the range of 800 to 4200 kcal for men and 500 to 3500 kcal for women) [38]. The UK Biobank study obtained ethics approval from the North West Multicenter Research Ethics Committee, and written informed consent was obtained from all participants [36].

### Exposure assessment

The Oxford WebQ is a questionnaire structured to record information about foods consumed by 21 food groups similar to the 24-hour-dietary recall [37], which has been validated in previous research [39]. The consumption of sugar and sugar subtypes was calculated using the Oxford WebQ, similar to previous studies [40, 41], between April 2009 and June 2012. The total energy and sugar contents were estimated for each Oxford WebQ questionnaire item based on the UK Nutrient Databank. Free sugar was classified based on the Scientific Advisory Committee on Nutrition (SACN) in the UK: all monosaccharides and disaccharides added to food by manufacturers, cooks, or consumers and sugars such as honey and syrups that are naturally present in food. Nutrient estimation procedures were conducted based on Perez-Cornago, Pollard [42] the nutrient intake per 100 g, which was calculated for each food item in the questionnaire. For items that included a question on sugar, if “varied” was selected, then 1 tsp of sugar was assumed. The following sugar items were included in the current study: total sugar, glucose, fructose, maltose, and sucrose. For each participant, the intake (g/day) of each sugar subtype was recorded, and the intake was calculated as energy (kJ/day) by multiplying the intake in g/day by 17 kJ/g. Sugar subtype consumption in percentage of total energy (%E) is calculated according to [43]: $$\begin{aligned} \mathrm{Sugar}\;\mathrm{intake}\;\mathrm{in}\;\mathrm{kJ}/\mathrm{day}\;\mathrm{divided}\;\mathrm{by}\;\mathrm{total}\;\mathrm{energy}\;\mathrm{in}\;\mathrm{kJ}/\mathrm{day}\times100 \end{aligned}$$. A sub-analysis on the non-linear association between added sugar and GAD score was conducted to elicit the effects of added sugar that differentiate from naturally occurring sugars. As all participants completed at least two questionnaires, the mean percentage of energy intake from sugar was used for all analyses.

### Outcome measures

The UK Biobank introduced the Mental Health Questionnaire in 2017 to measure common mental health symptoms including the Generalized Anxiety Disorder-7 (GAD-7) questionnaire. The GAD-7 is a 7-item anxiety scale with a four-point ordinal scale with a score ranging from 0 to 21. The GAD-7 is commonly used in both clinical settings and research. In the current study, the prevalence of GAD was defined via an established cut-off (scores ≥ 10). The primary analysis was conducted using the continuous GAD-7 score. Previous studies have indicated that this cut-off has a sensitivity of 89% and specificity of 82% [44].

### Covariates

The covariates included alcohol use (never, previous, current), ethnicity (white, mixed, Asian, black, Chinese, others), general health rating (excellent, good, fair, poor), highest qualification (college or University, national exams at ages 17–18 years, national exams at age 16 years, secondary education, vocational qualification, and professional), BMI, smoking status (never, previous, current), diet variation (never, sometimes, often), long-standing illness (yes, no), cancer (yes, no), other medical conditions (yes, no), physical activity [sum metabolic equivalent task (MET)-min per week for all activity], the Townsend deprivation index, and the average intake of dietary fat, protein, and fiber.

### Statistical analysis

Sample characteristics were summarized using means and standard deviations (SD) or counts and percentages. Demographic characteristics were compared according to sex and anxiety status (GAD-7 ≥ 10). T-tests and chi-square tests were used to compare the differences in continuous variables and categorical variables, respectively. Given that there were up to 8 years of time variation between the exposure and outcome measure, the nonlinear relationship between each sugar variable and the GAD scores was examined using generalized additive models (GAMs) with covariates being controlled. GAMs offer a flexible modelling approach that accommodates a non-linear relationship between the response variable and the predictor variables by utilizing smooth curves, while also accounting for covariates. Given the complexity of dietary patterns, a linear model may not capture key aspects of the relationship. Therefore, GAMs have the potential to achieve a high level of model fit while maintaining a concise representation of the fitted curve. Smoothing parameters were selected using the restricted maximum likelihood method (REML) and the default option of ten basis functions to represent smooth terms. Each measure was modelled against a penalized regression spline function. The smooth curves from the GAMs presented nonlinear associations. All analyses were stratified by sex (male, female) and age group (≤ 45, 46 to 64, ≥ 65 years) to improve the robustness of the analyses. Significant non-linear relationships were further tested with various knots (k = 5, 10, 15, 20) in the GAM models. Missing values were imputed using multiple imputation by chain equation. All the data were analyzed with R version 4.2.2 with the “mgcv” package in R. Two-tailed *p*-values less than 0.05 were considered statistically significant.

## Results

### Demographic characteristics

A total of 84,087 participants from the UK Biobank study met the inclusion criteria. Individuals with extreme values of total energy intake (*n* = 562) and those without completion of the GAD-7 questionnaire (*n* = 42,172) were excluded. The final sample predominantly consisted of White ethnic participants (97.4%), with an average age of 56.01 years (SD = 7.69), and 43.4% were male. Among the included participants, 58.3% reported being nonsmokers, and 94.5% were identified as current alcohol users. Notably, 3.94% (*n* = 3,312) of the sample had anxiety (GAD-7 score ≥ 10), with a higher prevalence among females (4.70% versus 2.95% in males) (Table 1). Females had higher dietary intakes of total sugar, glucose, fructose, and sucrose. Most of the background and lifestyle characteristics differed between males and females with or without anxiety, except for the presence of cancer and total energy intake (*p* > 0.05).

**Table 1:** Descriptive statistics between males and females with or without anxiety (anxiety was classified as GAD-7≥ 10)

	Total (*n* = 84087)	Women without anxiety (*n* = 45325)	Women with anxiety (*n* = 2234)	Men without anxiety (*n* = 35450)	Men with anxiety (*n* = 1078)	*p*
Age, mean (SD)	56.01 (7.69)	55.54 (7.57)	53.28 (7.61)	56.87 (7.73)	53.21 (8.03)	< 0.001
Highest qualification (%)						< 0.001
College/ University	47,975 (52.8)	22,187 (51.6)	964 (46.1)	18,362 (54.9)	462 (47.0)	
Secondary/ Professional education	36,112 (47.2)	23,138 (48.4)	1128 (53.9)	17,088 (45.1)	616 (53.0)	
Ethnic (%)						< 0.001
White	81,652 (97.4)	44,019 (97.3)	2132 (95.8)	34,465 (97.6)	1036 (96.5)	
Others	2435 (2.6)	1306 (2.7)	102 (4.2)	985 (2.4)	42 (3.5)	
Smoker (%)						< 0.001
Never	49,052 (58.3)	28,245 (62.1)	1246 (55.7)	19,117 (53.8)	516 (47.7)	
Previous	29,629 (35.3)	14,772 (32.6)	782 (35.1)	13,649 (38.6)	426 (39.6)	
Current	5406 (6.4)	2380 (5.3)	206 (9.2)	2684 (7.6)	136 (12.7)	
Alcohol use (%)						< 0.001
Never	2287 (2.7)	1531 (3.3)	102 (4.6)	624 (1.8)	30 (2.8)	
Previous	2294 (2.7)	1247 (2.8)	96 (4.3)	893 (2.5)	58 (5.4)	
Current	79,506 (94.6)	42,547 (93.9)	2036 (91.1)	33,933 (95.7)	988 (91.8)	
Townsend Deprivation index, mean (SD)	-1.69 (2.81)	-1.66 (2.79)	-1.08 (3.01)	-1.78 (2.80)	-1.10 (3.19)	< 0.001
BMI, mean (SD)	26.54 (4.51)	26.08 (4.75)	26.51 (5.71)	27.08 (3.98)	28.21 (4.80)	< 0.001
Health rating (%)						< 0.001
Excellent/ Good	69,892 (83.1)	38,895 (85.8)	1460 (65.4)	28,947 (81.7)	590 (54.7)	
Fair / Poor	14,195 (16.9)	6430 (14.2)	774 (34.6)	6503 (18.3)	577 (45.3)	
Physical activity (MET/min), mean (SD)	2367.49 (2293.70)	2331.20 (2197.91)	2395.11 (2357.79)	2404.19 (2388.88)	2538.18 (2623.51)	< 0.001
Diet Variation (%)						< 0.001
Never	30,562 (36.3)	15,123 (33.4)	781 (35.0)	14,268 (40.2)	390 (36.2)	
Sometimes	47,324 (56.3)	26,894 (59.3)	1269 (56.8)	18,595 (52.5)	566 (52.5)	
Often	6201 (7.4)	3308 (7.3)	184 (8.2)	2587 (7.3)	122 (11.3)	
Long standing illness/ disability = Yes (%)	25,803 (30.6)	12,332 (27.2)	913 (40.8)	11,992 (33.8)	566 (52.5)	< 0.001
Cancer = Yes (%)	7472 (8.8)	4447 (9.8)	205 (9.2)	2729 (7.7)	91 (8.4)	0.766
Other medical condition = Yes (%)	18,371 (21.8)	9258 (20.4)	619 (27.7)	8114 (22.9)	380 (35.2)	< 0.001
Protein intake g/day (mean (SD)	80.53 (19.93)	76.54 (17.89)	76.49 (20.52)	85.74 (20.96)	85.88 (24.35)	< 0.001
Fat intake g/day (mean (SD)	73.57 (23.92)	69.12 (21.41)	70.90 (23.86)	79.20 (25.50)	81.13 (28.87)	< 0.001
Fiber intake g/day (mean (SD)	17.96 (5.71)	17.66 (5.47)	17.62 (6.37)	18.36 (5.92)	18.02 (6.47)	< 0.001
Energy intake / kJ, mean (SD)	8629.21 (2039.51)	8029.69 (1736.59)	8178.66 (1999.38)	9396.13 (2119.61)	9550.05 (2433.63)	0.052
GAD-7 Score mean (SD)	2.04 (3.27)	1.78 (2.32)	13.69 (3.11)	1.29 (2.04)	13.81 (3.13)	< 0.001
Energy % contributed by Dietary sugar, mean (SD)						
Glucose	5.29 (2.05)	5.56 (2.09)	5.39 (2.20)	4.95 (1.92)	4.84 (2.13)	< 0.001
Fructose	5.71 (2.45)	6.09 (2.47)	5.84 (2.63)	5.22 (2.33)	5.04 (2.54)	< 0.001
Maltose	1.23 (0.95)	0.92 (0.52)	0.99 (0.61)	1.63 (1.19)	1.68 (1.25)	< 0.001
Sucrose	9.11 (3.40)	9.16 (3.18)	9.77 (3.91)	8.99 (3.60)	9.69 (4.26)	< 0.001

*BMI* Body Mass Index, *GAD* Generalised Anxiety Disorder-7; Age, Townsend deprivation index, BMI and physical activities and GAD scores were presented in mean (SD); highest qualification, ethnicity, smoker, alcohol use, health rating, diet variation, long-standing illness/ disability, cancer, other medical condition were presented in n (%)

### Nonlinear relationship between the type of sugar and the GAD score

**Fig. 1:**
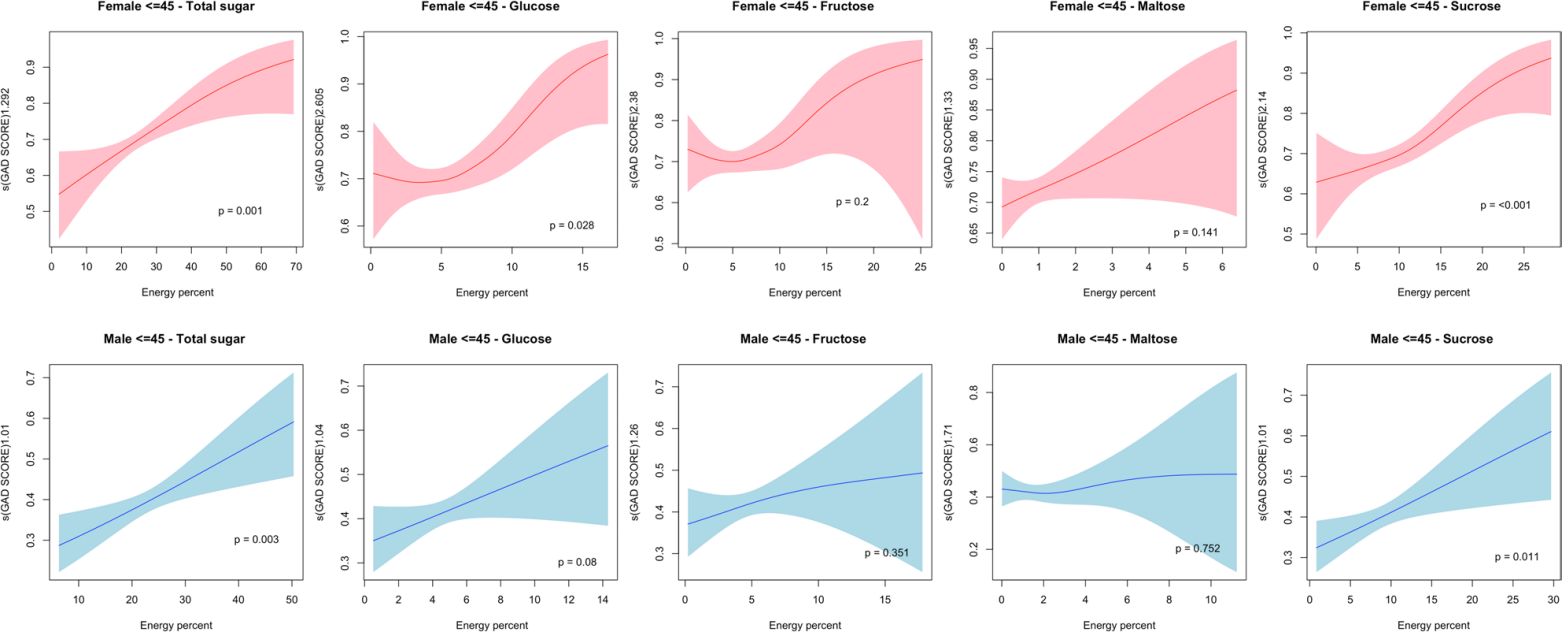
Non-linear relationship plots of sugar consumption and GAD score in males and females aged < = 45

Significant nonlinear relationships were found between different types of sugar consumption and GAD score, and the results were modified by sex and age. In those aged 45 years or younger, there were significant nonlinear associations between total sugar (*p* = 0.02)/sucrose (*p* = 0.011) consumption and the GAD score in males, and between total sugar (*p* = 0.001)/sucrose consumption (*p* < 0.001) and the GAD score in females. However, the shapes in Fig. 1 appeared to be monotonic. Nonlinear relationships of glucose (*p* = 0.028) and sucrose (*p* < 0.001) with significant thresholds of 5% and 10% respectively, increased GAD scores in females in this age group. The Wald test for interaction between gender and age group is shown in Appendix 1.

**Fig. 2:**
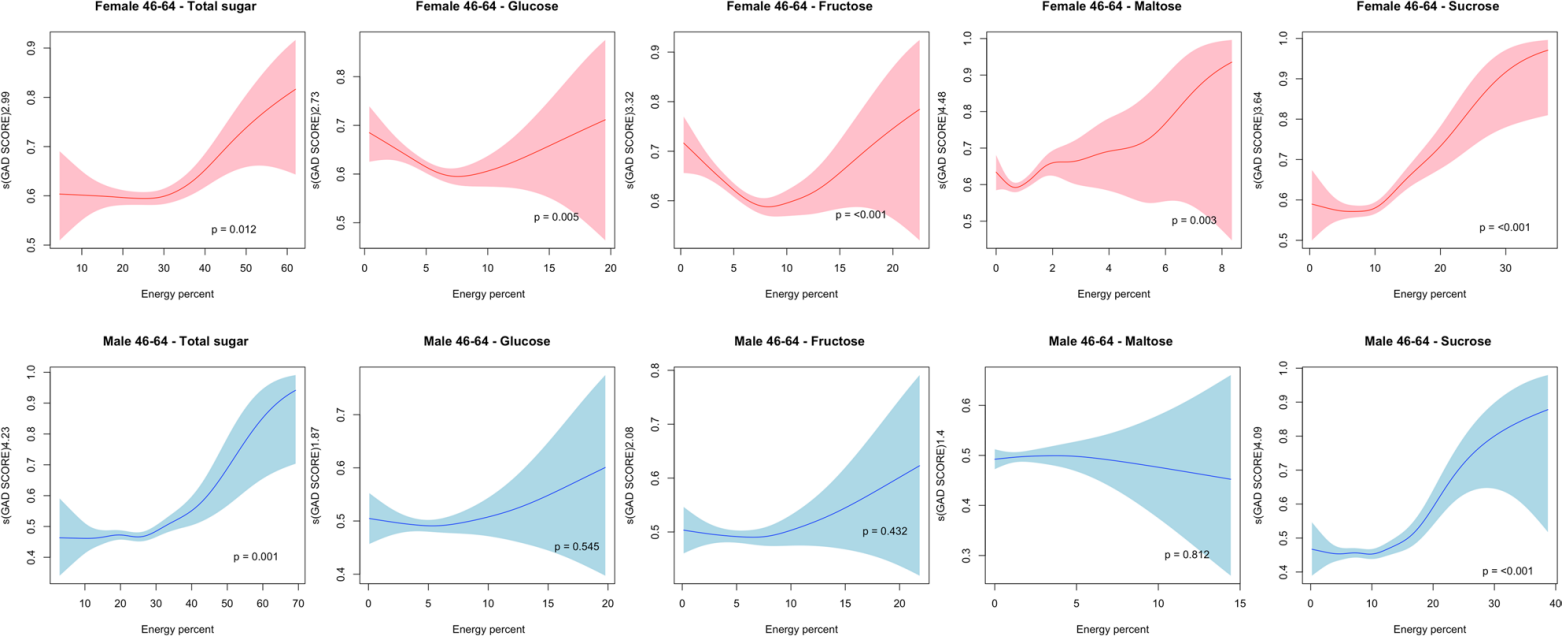
Non-linear relationship plot of sugar consumption and GAD score in males and females aged between 46 and 64

In the group aged 46 to 64 years (Fig. 2), nonlinear associations of GAD score with total sugar consumption were found for both genders (*p* < 0.01), where the consumption beyond 30% of the total energy intake significantly increased in GAD scores. Additionally, a nonlinear association was also observed across all the sugar types (*p* < 0.05) in females, whereas in males, only sucrose (*p* < 0.001) intake showed a significant nonlinear association. Specifically, the association between sucrose consumption and GAD score followed a J-shaped pattern in both genders; when intake exceeded the lowest point at approximately 10%, it significantly increased the GAD score. Other sugar types, including glucose (7%) and fructose (7%), demonstrated U-shaped associations in females only.

**Fig. 3:**
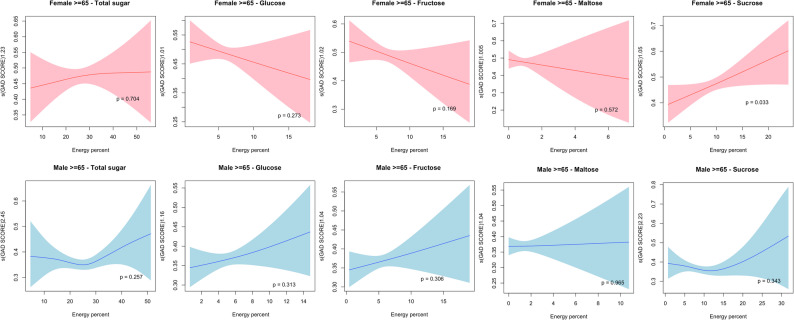
Non-linear relationship plots of sugar consumption and GAD score in males and females aged > = 65

For participants aged 65 or older, no significant nonlinear relationship was found in males. In females, only sucrose consumption demonstrated a nonlinear association with GAD score (*p* = 0.033). Nevertheless, the associations observed were monotonic (Fig. 3).

When investigating the non-linear associations between added sugar and GAD scores, our results revealed that in women across all age groups, added sugar was significantly associated with GAD scores. Meanwhile, in males, only individuals aged between 46 and 64 had demonstrated a significant non-linear association between added sugar and GAD score (Appendix 2).

Sensitivity of the significant non-linear GAM models is shown in Appendices 3 to 7. Overall, the sensitivity analysis revealed that across various knots in the GAM model, similar non-linear patterns were observed across various types of sugar associated with the GAD score.

## Discussion

This study was the first to explore the nonlinear association and thresholds of different types of sugar intake on anxiety symptoms according to age and sex using large-scale cohort data. We identified significant nonlinear roles of most sugar types in young women and all sugar types in middle-aged women. In contrast, the relationships tended to be monotonic in most age groups of men. Similar age/sex differences have also been reported in previous studies. For example, a cross-sectional study of 20,231 nondiabetic adults revealed that high-anxiety individuals aged under 45 years had significantly higher mean consumption of added simple sugars, but the difference was not significant in older individuals; women and younger individuals were more likely to have trait anxiety [10]. Our results showed that women were more likely to consume most types of sugars and experience anxiety. Unsurprisingly, women presented more nonlinear associations than men did, as suggested by previous research. Women tend to consume more added sugars than men and prefer snack-related foods (e.g., chocolate, ice cream) as comfort foods, whereas men prefer meal-related foods [45]. Women also tend to consume sugar as an emotional regulation strategy for stress and anxiety [46]. The nonlinear association may indicate the differences in stress and anxiety coping between men and women. Studies have shown that women are more likely to consume food in response to negative emotions [47, 48].

Furthermore, animal studies revealed that excessive consumption of sugar could promote neuroinflammation [25, 49]. Thus, low to moderate levels of sugar consumption may initially decrease anxiety symptoms. Meanwhile, as the quantity of sugar consumption becomes excessive, it worsens anxiety symptoms. As for the gender differences, these could potentially be influenced by various factors, including biological aspects (e.g., sex hormones, hormonal fluctuations throughout the menstrual cycle) [50, 51], cultural influences [52], and gender role expectations [53]. With respect to the age differences observed in our results, previous studies have shown that younger people and women are more likely to experience anxiety [54, 55] and that their emotional status and physio- and pathological status (e.g., hormones, gut microbiota, enterotypes, inflammation) may be more sensitive and responsive to sugar intake than older people and men. This could be due to differences in microbiota composition and microbiota changes throughout the lifespan, where patients with GAD exhibit an altered profile [56]. Ageing is also associated with a decline in GI function in nutrient digestion and absorption, which is related to the intestinal microbiota [57]. The differences in associations between age groups could indicate the significant role of the GI in anxiety symptoms.

Regarding the specific types of sugars, anxiety appeared to be more sensitive to the amount of sucrose consumed than to the amounts of glucose, fructose, or maltose. This may be due to their different chemical structures and properties. Sucrose is a common disaccharide composed of one glucose molecule and one fructose molecule joined together. It is commonly known as table sugar and is derived from sugarcane or sugar beets. Sucrose is widely used as a sweetener in various food and beverage products. Previous research suggested that because sucrose may have a direct effect on the brain via glucose and an effect on a peripheral mechanism via fructose, sucrose may have a stronger effect than only glucose on mood and cognitive performance [58]. Animal studies have revealed that the overconsumption of sucrose is linked to anxiety behaviours [20], the inhibition of opioid receptors in the brain [59], and the disruption of stress-coping mechanisms. However, intervention studies have revealed adverse effects or no beneficial effects of sucrose solutions on mood improvement, while several glucose interventions have shown small beneficial effects [19]. These short-term/immediate effects could be different from the long-term associations that we were testing in the survey. One-time sugar consumption and chronic sugar consumption habits may also lead to different consequences on anxiety and other health outcomes. The complex roles of different types of sugars under different conditions still need more experimental and longitudinal studies.

Both naturally occurring and added sugar may lead to a significant worsening of GAD symptoms. One of the major contributions is that we identified the thresholds of sugar consumption regarding their associations with anxiety. Limiting sugar consumption within 5% to 10% of the total energy intake would have no significant or even beneficial effect on anxiety, whereas more consumption beyond the cut-off would increase anxiety. This finding is consistent with the World Health Organization’s (WHO) recommendation that individuals reduce the intake of free sugars to < 10% of their daily energy intake, with a stricter target of less than 5% of daily energy intake for additional health benefits [7]. Our study extends its application to mental health by adding evidence on the relationships between these thresholds and anxiety.

These findings may have important implications for understanding lifestyle psychiatry, gut-brain connections, and improving health. Excessive sugar intake increases not only anxiety symptoms but also the risk of being overweight, obesity, nutritional deficiencies, type 2 diabetes, dental problems, cardiovascular diseases, chronic low-grade inflammation, and addiction, which may in turn contribute to one’s burden and anxiety. Thus, limiting sugar consumption under our recommended threshold, especially for younger women, will benefit various aspects of their health, help prevent future chronic diseases, and reduce the burden on the healthcare system. Longitudinal observations are therefore warranted to establish a causal relationship between sugar consumption and anxiety to increase awareness among the public regarding the harms of excessive sugar intake for emotional health and the recommended amount and threshold. Knowledge and skills related to reading food labels, choosing healthy foods, identifying low-sugar or sugar-free alternatives, and adopting adaptive stress-coping strategies should be enhanced.

### Limitations

Several limitations need to be acknowledged. First, the current study design is limited to cross-sectional and relies on the assumption that dietary patterns remain stable. The current study omitted the time-varying exposures and covariates because of the absence of repeated measurements in the UK Biobank. The assessment of dietary intake over a limited follow-up period only assesses a segment of individuals’ lifetime exposure. Additionally, the lack of a baseline anxiety measure limits the potential of a prospective assessment. Our results only rendered a cross-sectional analysis and should be interpreted with caution due to the potential for reciprocity. Secondly, this study’s reliance on the mean of only two 24-hour dietary recalls as a proxy for long-term habitual intake presents a limitation; the self-reported dietary recall may also increase the possibility of misreporting bias, as this method may not accurately capture the dietary patterns relevant to chronic conditions like mental health issues, thereby potentially introducing measurement error. Future studies should consider longitudinal designs to better capture the temporal relationship between dietary changes and mental health outcomes, thereby improving accuracy and validity. Third, utilisation of the GAD-7 questionnaire as an anxiety measure, which introduces subjectivity and has restricted accuracy. To investigate the immediate versus long-term effects of dietary sugar consumption, future studies using ecological momentary assessment (EMA) and panel study designs are needed. Fourth, we did not measure binge sugar intake or emotional eating as control variables. There is a possibility that high sugar consumption implies a case of binge sugar intake, which can cause overstimulation of dopamine in the brain. Studies have suggested that carbohydrate overconsumption stimulates the production and utilization of dopamine, similar to alcohol and other addictive substances [60]. Moreover, high sugar consumption may also be a result of [61] emotional eating, in which individuals cope with their stress and anxiety by eating. Future studies should test these possibilities to better understand the roles of sugar and anxiety.

In conclusion, the present research identified nonlinear associations and thresholds of various types of sugar on anxiety. Moreover, age and sex are demonstrated as moderators, particularly in females and middle-aged adults. Furthermore, sucrose consumption was most strongly associated with anxiety symptoms regardless of gender. Low to moderate sugar intake (e.g., short- versus long-term effects, one-off versus chronic intake) is recommended for emotional health. The complex roles of various types of sugar in emotional health should be better explored in future studies with more rigorous study designs.

## Supplementary Information

Supplementary Material 1: Appendix 1. Wald Test Results of the Generalized Additive Model of dietary sugar and GAD Scores. Appendix 2. Non-linear relationship plots of sugar consumption and GAD score in males and females. Appendix 3. Non-linear relationship plots of sugar consumption and GAD score in females aged <= 45. Appendix 4. Non-linear relationship plots of sugar consumption and GAD score in females aged between 46 and 64 years old. Appendix 5. Non-linear relationship plots of sugar consumption and GAD score in females aged >=65. Appendix 6. Non-linear relationship plots of sugar consumption and GAD score in males aged between <= 45. Appendix 7. Non-linear relationship plots of sugar consumption and GAD score in males aged between 46 and 64 years old.

## Data Availability

No datasets were generated or analysed during the current study.

## References

[1] WHO. ICD-11 for Mortality and Morbidity Statistics: World Health Organisation. 2023 [updated 01/2023. Available from: https://icd.who.int/browse11/l-m/en#/http%3a%2f%2fid.who.int%2ficd%2fentity%2f1336943699

[2] Collaborators GMD. Global, regional, and National burden of 12 mental disorders in 204 countries and territories, 1990–2019: a systematic analysis for the global burden of disease study 2019. Lancet Psychiatry. 2022;9(2):137–50. 35026139 10.1016/S2215-0366(21)00395-3 PMC8776563

[3] Szuhany KL, Simon NM. Anxiety disorders: A review. JAMA. 2022;328(24):2431–45. 36573969 10.1001/jama.2022.22744

[4] Konnopka A, Konig H. Economic burden of anxiety disorders: A systematic review and Meta-Analysis. PharmacoEconomics. 2020;38(1):25–37. 31646432 10.1007/s40273-019-00849-7

[5] Wittchen H-U, Mühlig S, Beesdo K. Mental disorders in primary care. Dialogues Clin Neurosci. 2003;2:115–28. 10.31887/DCNS.2003.5.2/huwittchen PMC3181625 22034245

[6] Castaldelli-Maia JM, Marziali ME, Lu Z, Martins SS. Investigating the effect of National government physical distancing measures on depression and anxiety during the COVID-19 pandemic through meta-analysis and meta-regression. Psychol Med. 2021;51(6):881–93. 33648613 10.1017/S0033291721000933 PMC7985907

[7] WHO. Guideline: sugars intake for adults and children. 2015. 25905159

[8] Wang X, Wong ACW, Sheng Z, Wong SY, Yang X. The relationship between dietary sugar consumption and anxiety disorders: A systematic review. Nutr Bull. 2024 Dec;49(4):429-443.10.1111/nbu.12702. Epub 2024 Aug 13. PMID: 39138127. 10.1111/nbu.12702 39138127

[9] Keck MM, Vivier H, Cassisi JE, Dvorak RD, Dunn ME, Neer SM, et al. Examining the role of anxiety and depression in dietary choices among college students. Nutrients. 2020;12(7):1–19. 10.3390/nu12072061 PMC7400947 32664465

[10] Kose J, Cheung A, Fezeu LK, Péneau S, Debras C, Touvier M, et al. A comparison of sugar intake between individuals with high and low trait anxiety: results from the nutrinet-santé study. Nutrients. 2021;13(5):1–11. 10.3390/nu13051526 PMC8147234 33946586

[11] Kose J, Fezeu LK, Touvier M, Peneau S, Hercberg S, Galan P, et al. Dietary macronutrient intake according to sex and trait anxiety level among non-diabetic adults: a cross-sectional study. Nutr J. 2021;20(1):78. 34496851 10.1186/s12937-021-00733-1 PMC8424616

[12] Kose J, Paz Graniel I, Péneau S, Julia C, Hercberg S, Galan P, et al. A population-based study of macronutrient intake according to mental health status with a focus on pure and comorbid anxiety and eating disorders. Eur J Nutr. 2022;61(7):3685–96. 35678893 10.1007/s00394-022-02923-x PMC9178539

[13] Gao C, Sun Y, Zhang F, Zhou F, Dong C, Ke Z, et al. Prevalence and correlates of lifestyle behavior, anxiety and depression in Chinese college freshman: A cross-sectional survey. Int J Nurs Sci. 2021;8(3):347–53. 34307785 10.1016/j.ijnss.2021.05.013 PMC8283720

[14] Liu J, Chen T, Chen M, Ma Y, Ma T, Gao D, et al. Sugar-Sweetened beverages and depressive and social anxiety symptoms among children and adolescents aged 7–17 Years, stratified by body composition. Front Nutr. 2022;9:888671. 35677554 10.3389/fnut.2022.888671 PMC9168881

[15] Shi Z, Taylor AW, Wittert G, Goldney R, Gill TK. Soft drink consumption and mental health problems among adults in Australia. Public Health Nutr. 2010;13(7):1073–9. 20074392 10.1017/S1368980009993132

[16] Vassou C, Yannakoulia M, Georgousopoulou EN, Pitsavos C, Cropley M, Panagiotakos DB. Foods, Nutrients and Dietary Patterns in Relation to Irrational Beliefs and Related Psychological Disorders: The ATTICA Epidemiological Study. Nutrients. 2021 Apr 27;13(5):1472. 10.3390/nu13051472. PMID: 33925406; PMCID: PMC8146573. 10.3390/nu13051472 PMC8146573 33925406

[17] Sangsefidi ZS, Lorzadeh E, Hosseinzadeh M, Mirzaei M. Dietary habits and psychological disorders in a large sample of Iranian adults: A population-based study. Ann Gen Psychiatry. 2020;19(1):1–10. 32123535 10.1186/s12991-020-00263-w PMC7041096

[18] Firth J, Solmi M, Wootton RE, Vancampfort D, Schuch FB, Hoare E, et al. A meta-review of lifestyle psychiatry: the role of exercise, smoking, diet and sleep in the prevention and treatment of mental disorders. World Psychiatry. 2020;19(3):360–80. 32931092 10.1002/wps.20773 PMC7491615

[19] van de Rest O, van der Zwaluw NL, de Groot L. Effects of glucose and sucrose on mood: a systematic review of interventional studies. Nutr Rev. 2018;76(2):108–16. 29228399 10.1093/nutrit/nux065

[20] Jacques A, Chaaya N, Beecher K, Ali SA, Belmer A, Bartlett S. The impact of sugar consumption on stress driven, emotional and addictive behaviors. Neurosci Biobehav Rev. 2019;103:178–99. 31125634 10.1016/j.neubiorev.2019.05.021

[21] Javadi Arjmand E, Bemanian M, Vold JH, Skogen JC, Sandal GM, Arnesen EK, Mæland S, Fadnes LT. Emotional Eating and Changes in High-Sugar Food and Drink Consumption Linked to Psychological Distress and Worries: A Cohort Study from Norway. Nutrients. 2023 Feb 2;15(3):778. 10.3390/nu15030778. PMID: 36771484; PMCID: PMC9920951. 10.3390/nu15030778 PMC9920951 36771484

[22] Ulrich-Lai YM. Self-medication with sucrose. Curr Opin Behav Sci. 2016;9:78–83. 26977424 10.1016/j.cobeha.2016.02.015 PMC4787559

[23] Freeman CR, Zehra A, Ramirez V, Wiers CE, Volkow ND, Wang G-J. Impact of sugar on the body, brain, and behavior. Front Biosci (Landmark Ed). 2018;23(12):2255–66. 29772560 10.2741/4704

[24] Mergenthaler P, Lindauer U, Dienel GA, Meisel A. Sugar for the brain: the role of glucose in physiological and pathological brain function. Trends Neurosci. 2013;36(10):587–97. 23968694 10.1016/j.tins.2013.07.001 PMC3900881

[25] Ma X, Nan F, Liang H, Shu P, Fan X, Song X, et al. Excessive intake of sugar: an accomplice of inflammation. Front Immunol. 2022;13:988481. 36119103 10.3389/fimmu.2022.988481 PMC9471313

[26] Harrell CS, Burgado J, Kelly SD, Johnson ZP, Neigh GN. High-fructose diet during periadolescent development increases depressive-like behavior and remodels the hypothalamic transcriptome in male rats. Psychoneuroendocrinology. 2015;62:252–64. 26356038 10.1016/j.psyneuen.2015.08.025 PMC4637272

[27] de Sousa Rodrigues ME, Bekhbat M, Houser MC, Chang J, Walker DI, Jones DP, et al. Chronic psychological stress and high-fat high-fructose diet disrupt metabolic and inflammatory gene networks in the brain, liver, and gut and promote behavioral deficits in mice. Brain Behav Immun. 2017;59:158–72. 27592562 10.1016/j.bbi.2016.08.021 PMC5154856

[28] Rodgers B, Korten AE, Jorm AF, Jacomb PA, Christensen H, Henderson AS. Non-linear relationships in associations of depression and anxiety with alcohol use. Psychol Med. 2000;30(2):421–32. 10824662 10.1017/s0033291799001865

[29] Power C, Rodgers B, Hope S. U-shaped relation for alcohol consumption and health in early adulthood and implication for mortality. Lancet. 1998;352(9131):877. 9742982 10.1016/S0140-6736(98)23937-7

[30] Heilig M, Goldman D, Berrettini W, O’Brien CP. Pharmacogenetic approaches to the treatment of alcohol addiction. Nat Rev Neurosci. 2011;12(11):670–84. 22011682 10.1038/nrn3110 PMC3408029

[31] Mohler H. The GABA system in anxiety and depression and its therapeutic potential. Neuropharmacology. 2012;62(1):42–53. 21889518 10.1016/j.neuropharm.2011.08.040

[32] Huang Y, Chen Z, Chen B, Li J, Yuan X, Li J, et al. Dietary sugar consumption and health: umbrella review. BMJ. 2023;381:e071609. 37019448 10.1136/bmj-2022-071609 PMC10074550

[33] Azais-Braesco V, Sluik D, Maillot M, Kok F, Moreno LA. A review of total & added sugar intakes and dietary sources in Europe. Nutr J. 2017;16(1):6. 28109280 10.1186/s12937-016-0225-2 PMC5251321

[34] Michels, N. Poor Mental Health Is Related to Excess Weight via Lifestyle: A Cross-Sectional Gender- and Age-Dependent Mediation Analysis. Nutrients 2021;13:406. 10.3390/nu13020406. 10.3390/nu13020406 PMC7912087 33525320

[35] Lee J, Allen J. Gender differences in healthy and unhealthy food consumption and its relationship with depression in young adulthood. Community Ment Health J. 2021;57(5):898–909. 32602082 10.1007/s10597-020-00672-x

[36] Biobank U. UK Biobank: Protocol for a large-scale pro- spective epidemiological resource 2007. Available from: https://www.ukbiobank.ac.uk/media/gnkeyh2q/study-rationale.pdf.

[37] Liu B, Young H, Crowe FL, Benson VS, Spencer EA, Key TJ, et al. Development and evaluation of the Oxford WebQ, a low-cost, web-based method for assessment of previous 24 h dietary intakes in large-scale prospective studies. Public Health Nutr. 2011;14(11):1998–2005. 21729481 10.1017/S1368980011000942

[38] Kelly RK, Watling CZ, Tong TYN, Piernas C, Carter JL, Papier K, Arteriosclerosis, et al. Thromb Vascular Biology. 2021;4(1):2190–200. 10.1161/ATVBAHA.120.315628 PMC8216602 34039019

[39] Greenwood DC, Hardie LJ, Frost GS, Alwan NA, Bradbury KE, Carter M, et al. Validation of the Oxford WebQ online 24-Hour dietary questionnaire using biomarkers. Am J Epidemiol. 2019;188(10):1858–67. 31318012 10.1093/aje/kwz165 PMC7254925

[40] Kaiser A, Schaefer SM, Behrendt I, Eichner G, Fasshauer M. Association of sugar intake from different sources with incident depression in the prospective cohort of UK biobank participants. Eur J Nutr. 2023;62(2):727–38. 36205767 10.1007/s00394-022-03022-7 PMC9941260

[41] Behrendt I, Fasshauer M, Eichner G. Gluten intake and metabolic health: conflicting findings from the UK biobank. Eur J Nutr. 2021;60(3):1547–59. 32761538 10.1007/s00394-020-02351-9 PMC7987594

[42] Perez-Cornago A, Pollard Z, Young H, van Uden M, Andrews C, Piernas C, et al. Description of the updated nutrition calculation of the Oxford WebQ questionnaire and comparison with the previous version among 207,144 participants in UK biobank. Eur J Nutr. 2021;60(7):4019–30. 33956230 10.1007/s00394-021-02558-4 PMC8437868

[43] Willett WC, Howe GR, Kushi LH. Adjustment for total energy intake in epidemiologic studies. Am J Clin Nutr. 1997;65(4 Suppl):S1220–8. discussion 9S-31S. 10.1093/ajcn/65.4.1220S 9094926

[44] Spitzer R, Kroenke K, Williams J, Löwe B. A brief measure for assessing generalized anxiety disorder. Arch Intern Med. 2006;166(10):1092–7. 16717171 10.1001/archinte.166.10.1092

[45] Wansink B, Cheney MM, Chan N. Exploring comfort food preferences across age and gender. Physiol Behav. 2003;79(4–5):739–47. 12954417 10.1016/s0031-9384(03)00203-8

[46] Bekker MHJ. Janneke vM-V. Anxiety disorders: sex differences in Prevalence, Degree, and Background, but Gender-Neutral treatment. Gend Med. 2007;4:178–93. 10.1016/s1550-8579(07)80057-x 18156102

[47] Matud MP. Gender differences in stress and coping styles. Pers Indiv Differ. 2004;37(7):1401–15.

[48] Guerrero-Hreins E, Stammers L, Wong L, Brown RM, Sumithran P. A comparison of emotional triggers for eating in men and women with obesity. Nutrients. 2022;14:19. 10.3390/nu14194144 PMC9570591 36235796

[49] Gomes JAS, Silva JF, Marcal AP, Silva GC, Gomes GF, de Oliveira ACP, et al. High-refined carbohydrate diet consumption induces neuroinflammation and anxiety-like behavior in mice. J Nutr Biochem. 2020;77:108317. 32004874 10.1016/j.jnutbio.2019.108317

[50] Maeng LY, Milad MR. Sex differences in anxiety disorders: interactions between fear, stress, and gonadal hormones. Horm Behav. 2015;76:106–17. 25888456 10.1016/j.yhbeh.2015.04.002 PMC4823998

[51] Li SH, Graham BM. Why are women so vulnerable to anxiety, trauma-related and stress-related disorders? The potential role of sex hormones. Lancet Psychiatry. 2017;4(1):73–82. 27856395 10.1016/S2215-0366(16)30358-3

[52] Hofmann SG, Hinton DE. Cross-cultural aspects of anxiety disorders. Curr Psychiatry Rep. 2014;16(6):450. 24744049 10.1007/s11920-014-0450-3 PMC4037698

[53] Stoyanova M, Hope DA. Gender, gender roles, and anxiety: perceived confirmability of self report, behavioral avoidance, and physiological reactivity. J Anxiety Disord. 2012;26(1):206–14. 22154338 10.1016/j.janxdis.2011.11.006

[54] Hobbs MJ, Anderson TM, Slade T, Andrews G. Relationship between measurement invariance and age-related differences in the prevalence of generalized anxiety disorder. J Affect Disord. 2014;152–154:306–12. 24148792 10.1016/j.jad.2013.09.030

[55] Schieman S, AGE, PHYSICAL IMPAIRMENT, AND SYMPTOMS OF ANXIETY:. A TEST OF MEDIATING AND MODERATING FACTORS*. Int J Aging Hum Dev. 1999;49(1):43–59. 10614832 10.2190/P4EA-5CQ5-NMHD-30BX

[56] Kumar, A.; Pramanik, J.; Goyal, N.; Chauhan, D.; Sivamaruthi, B.S.; Prajapati, B.G.; Chaiyasut, C. Gut Microbiota in Anxiety and Depression: Unveiling the Relationships and Management Options. Pharmaceuticals 2023;16:565. 10.3390/ph16040565. 10.3390/ph16040565 PMC10146621 37111321

[57] An R, Wilms E, Masclee AAM, Smidt H, Zoetendal EG, Jonkers D. Age-dependent changes in GI physiology and microbiota: time to reconsider? Gut. 2018;67(12):2213–22. 30194220 10.1136/gutjnl-2017-315542

[58] van der Zwaluw NL, van de Rest O, Kessels RP, de Groot LC. Short-term effects of glucose and sucrose on cognitive performance and mood in elderly people. J Clin Exp Neuropsychol. 2014;36(5):517–27. 24839862 10.1080/13803395.2014.912613

[59] Colantuoni C, Schwenker J, McCarthy J, Rada P, Ladenheim B, Cadet J-L et al. Excessive sugar intake alters binding to dopamine and mu-opioid receptors in the brain. MOTIVATION, EMOTION, FEEDING, DRINKING. 2011;12(16):3549-52. 10.1097/00001756-200111160-00035 11733709

[60] Blum K, Braverman ER, Holder JM, Lubar JF, Monastra VJ, Miller D, Lubar JO, Chen TJ, Comings DE. Reward deficiency syndrome: a biogenetic model for the diagnosis and treatment of impulsive, addictive, and compulsive behaviors. J Psychoactive Drugs. 2000 Nov;32 Suppl:i-iv, 1-112. 10.1080/02791072.2000.10736099. PMID: 11280926. 10.1080/02791072.2000.10736099 11280926

[61] Braden A, Musher-Eizenman D, Watford T, Emley E. Eating when depressed, anxious, bored, or happy: are emotional eating types associated with unique psychological and physical health correlates? Appetite. 2018;125:410–7. 29476800 10.1016/j.appet.2018.02.022

