# Supplementary material for: Examining the non-linear relationship between sugar consumption and anxiety symptoms in UK biobank data
Source: Nutr J. 2026 Jan 26;25:25. doi: 10.1186/s12937-025-01277-4 (PMC12918462; doi:10.1186/s12937-025-01277-4)
Supplement: Supplementary file 1 — Supplementary Material 1: Appendix 1. Wald Test Results of the Generalized Additive Model of dietary sugar and GAD Scores. Appendix 2. Non-linear relationship plots of sugar consumption and GAD score in males and females. Appendix 3. Non-linear relationship plots of sugar consumption and GAD score in females aged <= 45. Appendix 4. Non-linear relationship plots of sugar consumption and GAD score in females aged between 46 and 64 years old. Appendix 5. Non-linear relationship plots of sugar consumption and GAD score in females aged >=65. Appendix 6. Non-linear relationship plots of sugar consumption and GAD score in males aged between <= 45. Appendix 7. Non-linear relationship plots of sugar consumption and GAD score in males aged between 46 and 64 years old. [file 12937_2025_1277_MOESM1_ESM.docx]

Appendix

Appendix 1. Wald Test Results of the Generalized Additive Model of dietary sugar and GAD Scores

| Total Sugar | Estimate | Standard Error | t-value | p-value |
| --- | --- | --- | --- | --- |
| <=45: Females | Reference | - | - | - |
| 46 to 64: Female | **1.59** | 0.740 | 2.154 | 0.031 |
| >= 65: Female | **1.84** | 0.807 | 2.273 | 0.023 |
| Glucose |  |  |  |  |
| <=45: Females | Reference | - | - | - |
| 46 to 64: Female | **1.56** | 0.740 | 2.108 | 0.035 |
| >= 65: Female | **1.78** | 0.807 | 2.206 | 0.028 |
| Fructose |  |  |  |  |
| <=45: Females | Reference | - | - | - |
| 46 to 64: Female | **1.55** | 0.739 | 2.102 | 0.036 |
| >= 65: Female | **1.77** | 0.807 | 2.197 | 0.028 |
| Maltose |  |  |  |  |
| <=45: Females | Reference | - | - | - |
| 46 to 64: Female | **1.54** | 0.739 | 2.084 | 0.037 |
| >= 65: Female | **1.79** | 0.806 | 2.215 | 0.027 |
| Sucrose |  |  |  |  |
| <=45: Females | Reference | - | - | - |
| 46 to 64: Female | **1.605** | 0.740 | 2.169 | 0.03 |
| >= 65: Female | **1.818** | 0.806 | 2.256 | 0.024 |
| Added Sugar |  |  |  |  |
| <=45: Females | Reference | - | - | - |
| 46 to 64: Female | **1.574** | 0.739 | 2.129 | 0.033 |
| >= 65: Female | **1.792** | 0.806 | 2.222 | 0.026 |

Appendix 2. Non-linear relationship plots of sugar consumption and GAD score in males and females

| 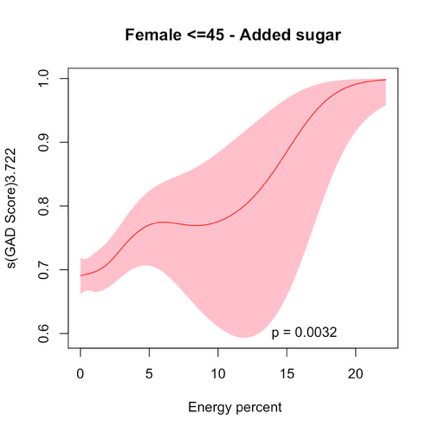 | 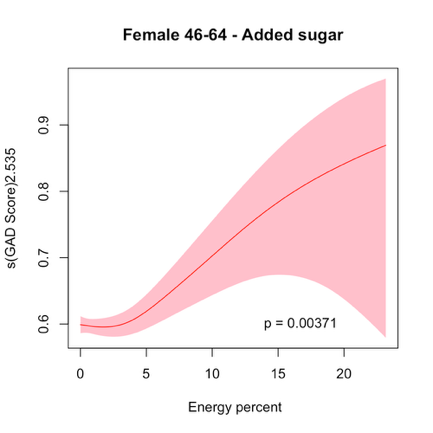 | 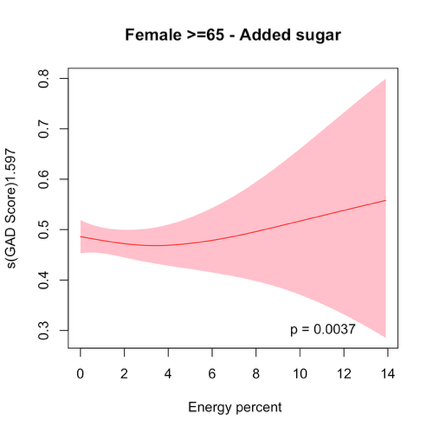 |
| --- | --- | --- |
| 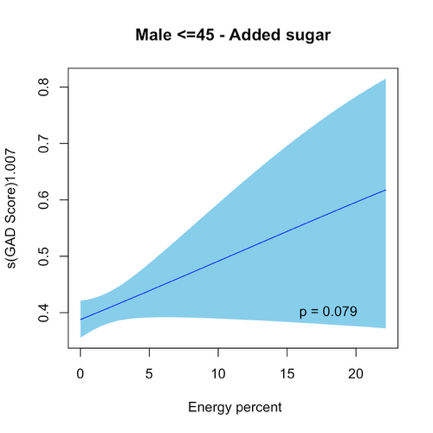 | 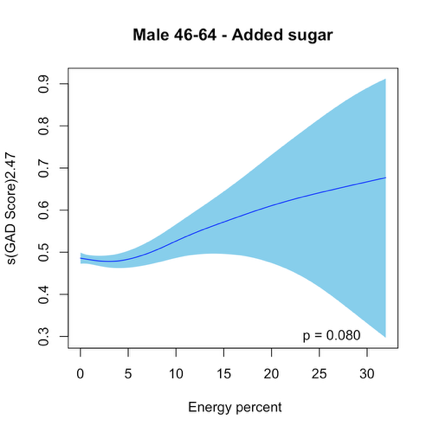 | 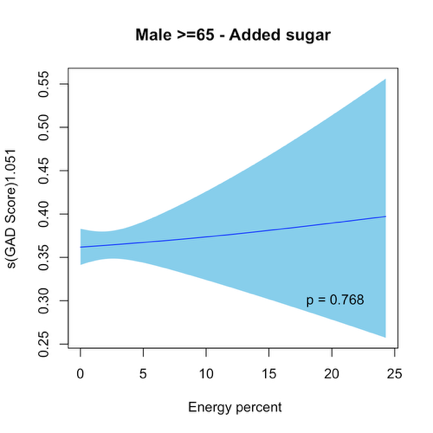 |


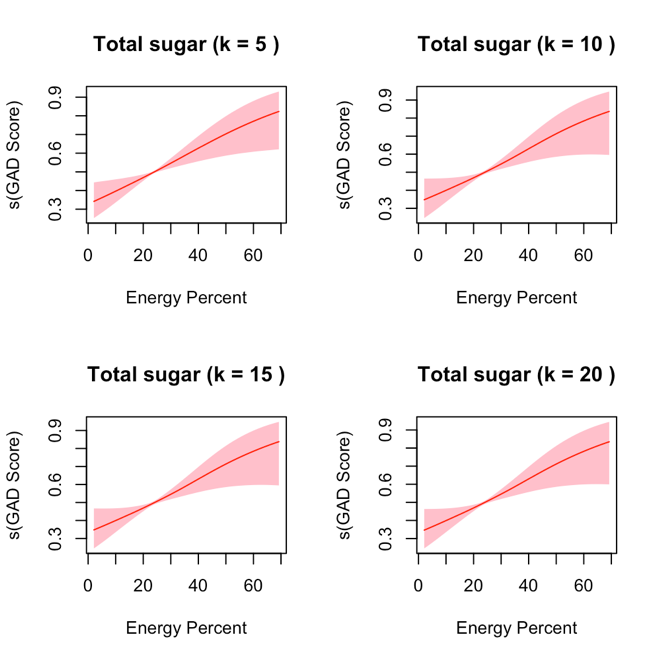

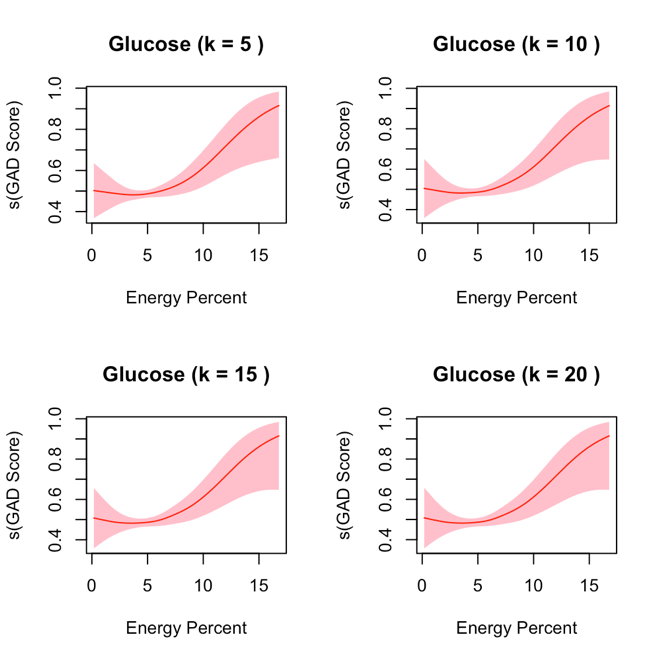

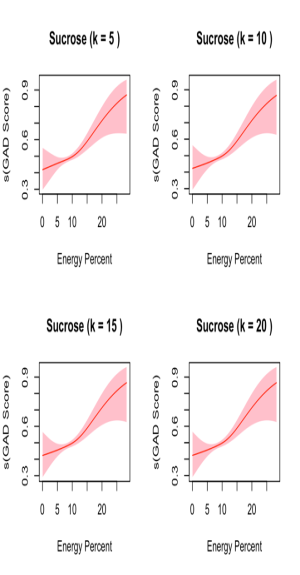


Appendix 3. Non-linear relationship plots of sugar consumption and GAD score in females aged <= 45


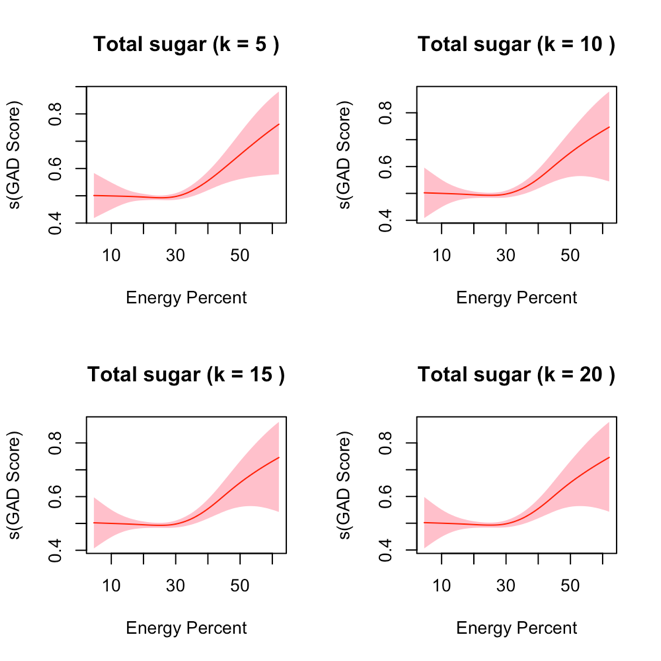

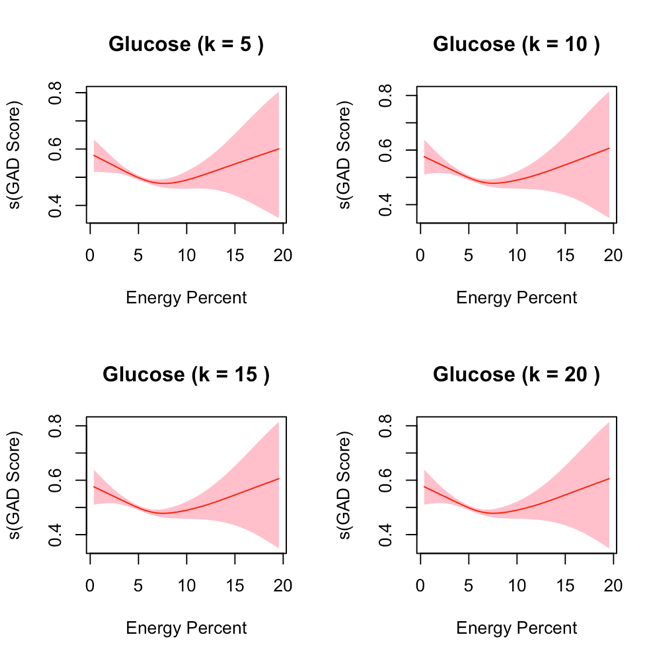

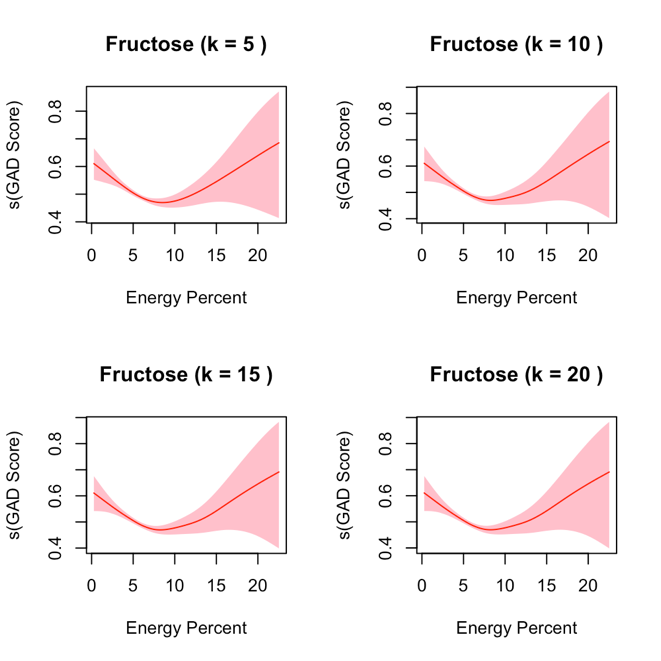

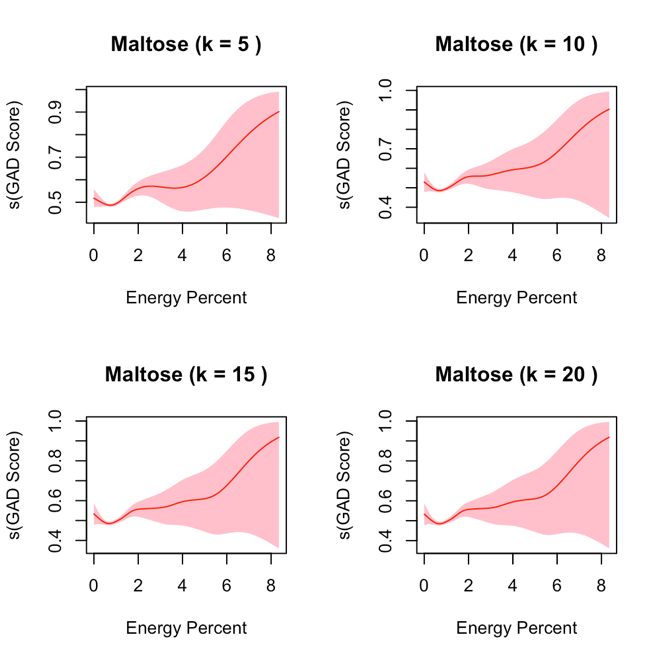

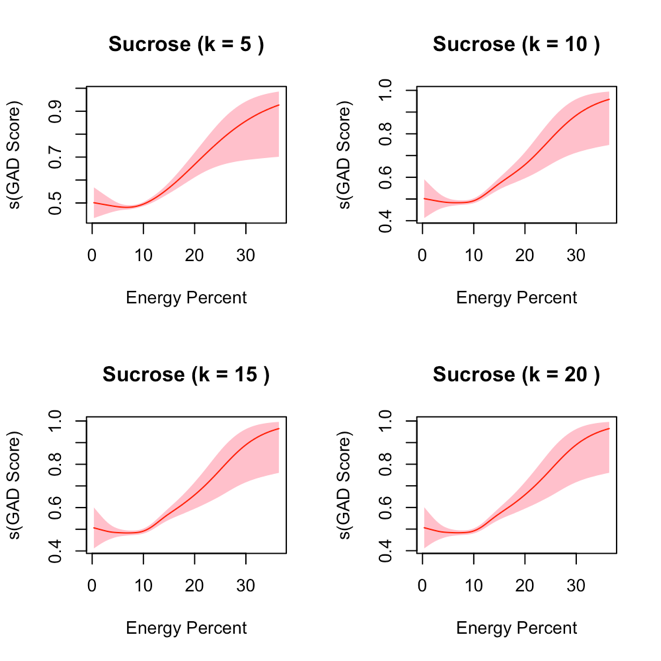


Appendix 4. Non-linear relationship plots of sugar consumption and GAD score in females aged between 46 and 64 years old


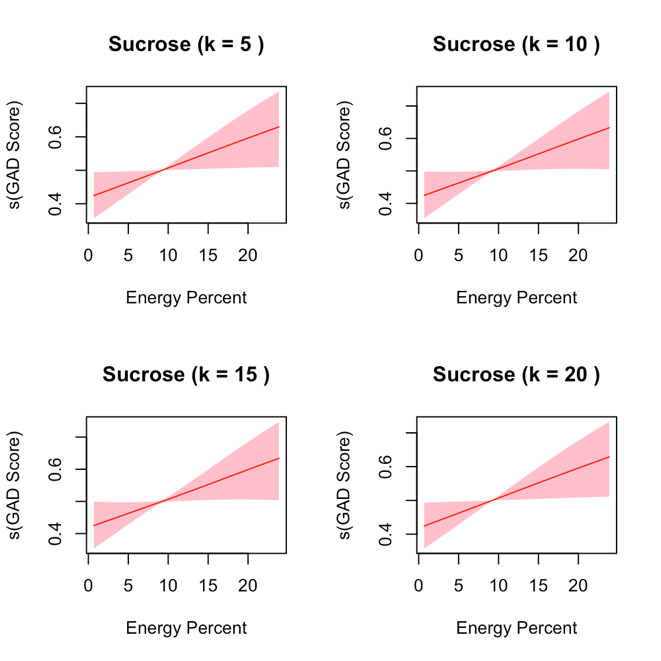


Appendix 5. Non-linear relationship plots of sugar consumption and GAD score in females aged >=65


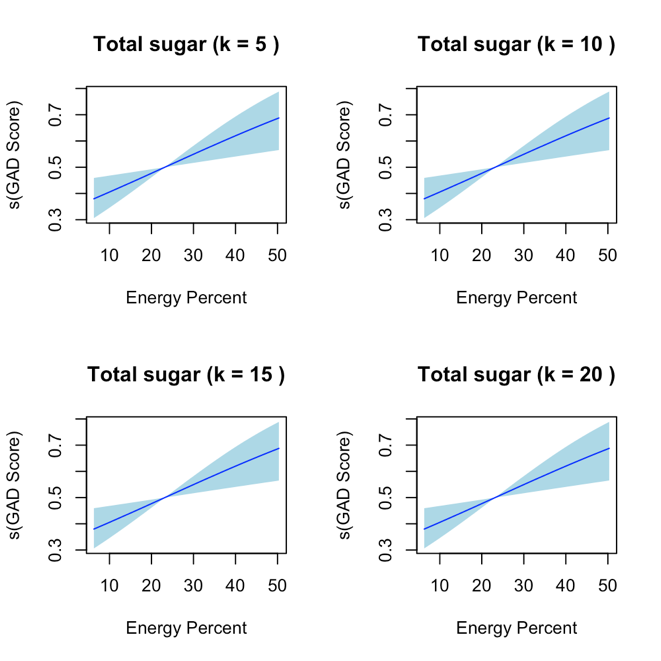

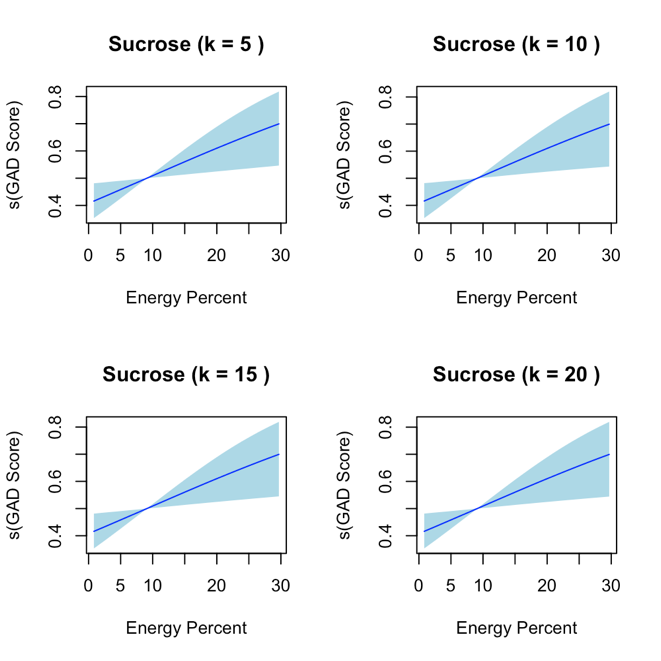


Appendix 6. Non-linear relationship plots of sugar consumption and GAD score in males aged between <= 45


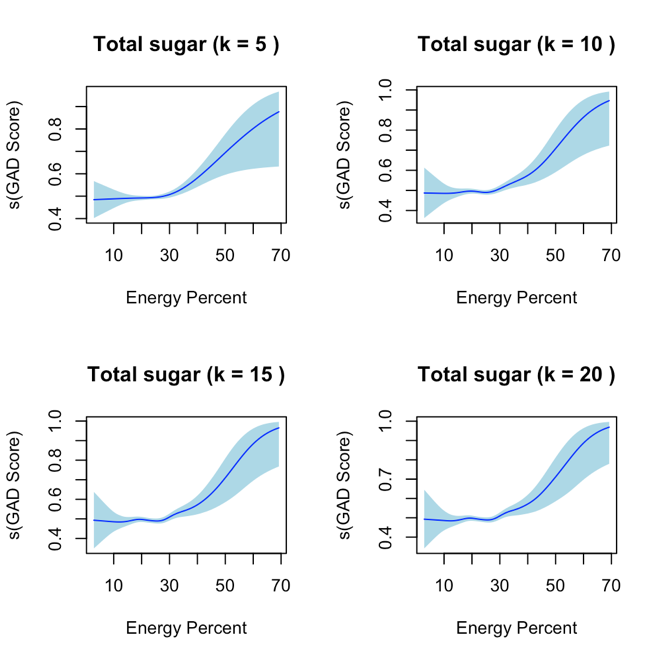

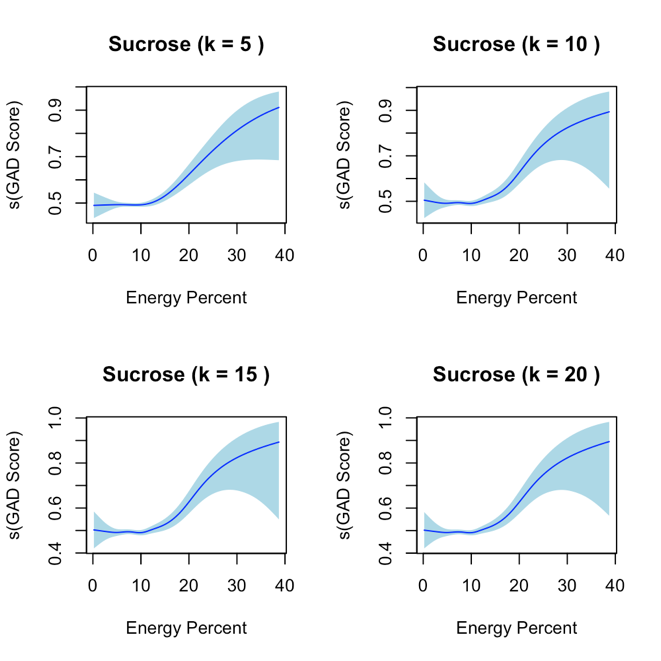


Appendix 7. Non-linear relationship plots of sugar consumption and GAD score in males aged between 46 and 64 years old
